# Analysis in *Proceratophrys boiei* genome illuminates the satellite DNA content in a frog from the Brazilian Atlantic forest

**DOI:** 10.3389/fgene.2023.1101397

**Published:** 2023-03-29

**Authors:** Marcelo João Da Silva, Thiago Gazoni, Célio Fernando Baptista Haddad, Patricia Pasquali Parise-Maltempi

**Affiliations:** ^1^ Departamento de Biologia Geral e Aplicada, Instituto de Biociências (IB), Universidade Estadual Paulista (UNESP), São Paulo, Brazil; ^2^ Departamento de Biodiversidade e Centro de Aquicultura, Instituto de Biociências (IB), Universidade Estadual Paulista (UNESP), São Paulo, Brazil

**Keywords:** repetitive DNA, satellitome, cytogenetics, evolution, cytogenomics

## Abstract

Satellite DNAs (satDNAs) are one of the most abundant elements in genomes. Characterized as tandemly organized sequences that can be amplified into multiple copies, mainly in heterochromatic regions. The frog *P. boiei* (2n = 22, ZZ♂/ZW♀) is found in the Brazilian Atlantic forest and has an atypical pattern of heterochromatin distribution when compared to other anuran amphibians, with large pericentromeric blocks on all chromosomes. In addition, females of *Proceratophrys boiei* have a metacentric sex chromosome W showing heterochromatin in all chromosomal extension. In this work, we performed high-throughput genomic, bioinformatic, and cytogenetic analyses to characterize the satellite DNA content (satellitome) in *P. boiei*, mainly due to high amount of C-positive heterochromatin and the highly heterochromatic W sex chromosome. After all the analyses, it is remarkable that the satellitome of *P. boiei* is composed of a high number of satDNA families (226), making *P. boiei* the frog species with the highest number of satellites described so far. Consistent with the observation of large centromeric C-positive heterochromatin blocks, the genome of *P. boiei* is enriched with high copy number of repetitive DNAs, with total satDNA abundance comprising 16.87% of the genome. We successfully mapped *via* Fluorescence in situ hybridization the two most abundant repeats in the genome, PboSat01-176 and PboSat02-192, highlighting the presence of certain satDNAs sequences in strategic chromosomal regions (e.g., centromere and pericentromeric region), which leads to their participation in crucial processes for genomic organization and maintenance. Our study reveals a great diversity of satellite repeats that are driving genomic organization in this frog species. The characterization and approaches regarding satDNAs in this species of frog allowed the confirmation of some insights from satellite biology and a possible relationship with the evolution of sex chromosomes, especially in anuran amphibians, including *P. boiei*, for which data were not available.

## 1 Introduction

Most eukaryotic genomes contain large blocks of heterochromatin surrounding centromeres and telomeres (Plohl et al., 2012; Garrido-Ramos, 2017; Sneideman and Meller, 2021), which are regions mainly composed of satellite DNAs repeats (satDNAs) and transposable elements (TEs). It is already known that satellite repeats make up sizable portions of genomes. SatDNAs participate in several processes, including gene regulation, stress response, and nuclear organization in many different organisms, making them prominent actors in the evolution of genomes, mainly because of their high plasticity (Plohl et al., 2012; Garrido-Ramos, 2017; Sneideman and Meller, 2021; Thakur et al., 2021).

Among vertebrates, amphibians are the class with the greatest variety of genome sizes (Liedtke et al., 2018; Gregory, 2022). Analysis of genomic sequencing reads combined with computer programs [e.g., RepeatExplorer (Novák et al., 2013)] have shown potential in the characterization of repetitive sequences in different organisms, including amphibians (Palacios-Gimenez et al., 2017; Silva et al., 2017; Ruiz-Ruano et al., 2018; Utsunomia et al., 2019; Da Silva et al., 2020; Ferretti et al., 2020; Crepaldi et al., 2021). Prior to that, with limitations, genomic digestion by restriction endonucleases was successfully used to characterize repetitive sequences in amphibians, mainly satDNAs, such as the PcP190 satellite in Hyloidea and Hylidae (Vittorazzi et al., 2011, 2014; Gatto et al., 2019, respectively) and more recently, the BamHI-800 satellite, a new satDNA family in Bufonidae (Guzmán et al., 2022).

The variability of satellite repeats is very interesting from an evolutionary point of view, as repeats within the same satDNA family evolve non-independently, showing low rates of divergence between monomers, through a process known as “concerted evolution” (Dover, 1982; Elder and Turner, 1995; reviewed by Thakur et al. (2021)). The concerted evolution occurs *via* “molecular drive”, an evolutionary process emerging from the activities of a number of ubiquitous mechanisms of DNA turnover, such as gene conversion, unequal crossing over, replication slippage, rolling circle replication, and multiple TE insertions (Dover, 1982; Dover, 1986; Elder and Turner, 1995; Ugarković and Plohl, 2002; Thakur et al., 2021). However, there are levels of variation in the rates of expansion, homogenization, and fixation between sequences. These dynamics depend on several factors, such as mutation rate, array size and structure, chromosome structure, and recombination rates (López-Flores and Garrido-Ramos, 2012; Plohl et al., 2012; Garrido-Ramos, 2017).

In genomes, sex chromosomes frequently accumulate satDNAs. Due to their differentiated and highly heterochromatic nature, sex chromosomes are a good example of genomic entities with expansions and contractions of heterochromatin throughout evolution. The gain of repetitive sequences, such as satDNA families, contributes to gradually differentiate from their homologs becoming heteromorphic sex chromosomes, which carry rapid diversification (Chalopin et al., 2015; Palacios-Gimenez et al., 2015; Wright et al., 2016; Yano et al., 2017; Sember et al., 2018; Charlesworth, 2021; Kratochvíl et al., 2021). Heteromorphic sex chromosomes show signs of degeneration such as extensive accumulation of transposable elements and other repeats, resulting in an enlargement of the sex-limited chromosomes (Y or W). Increased heterochromatinization or a diminishment of their size and gene loss are consequences of the long-term recombination suppression between the sex chromosomes (Charlesworth, 1991; Bergero and Charlesworth, 2009; Bachtrog et al., 2011; Schartl et al., 2016).

Sex chromosomes have independently evolved multiple times and show varied levels of divergence from each other in the heterogametic sex in XY males or ZW females (Bachtrog, 2008; Bachtrog et al., 2014). Unlike other organisms, like mammalians, birds, and insects, the majority of amphibian species (∼75%) has homomorphic sex chromosomes with both male and female heterogamy cases (Graves, 2008; Schmid et al., 2015; Ma and Veltsos, 2021), but a higher male heterogametic occurrence (26.7%, XX/XY) than female (8.8%, ZW/ZZ) system. About 41.5% of these species have an unknown system [Reviewed by Ma and Veltsos (2021)] and there are specific groups with unique sex chromosome systems, such as *Leptodactylus pentadactylus* (X1Y1X2Y2X3Y3X4Y4X5Y5X6Y6) and *Leiopelma hochstetteri* (WO/OO) (Green et al., 1993; Gazoni et al., 2018). These characteristics make frogs an interesting system to study sex chromosome diversity and evolution, as they harbor multiple stages of differentiation, with diverse sex determination system across species, as well as between and within populations of the same species (Nakamura, 2009; Malcom et al., 2014; Perrin, 2020; Ma and Veltsos, 2021).

Surprisingly, the Brazilian frog *P. boiei* (2n = 22, ZZ♂/ZW♀) has an atypical pattern of heterochromatin distribution, when compared to other anuran amphibians, with large pericentromeric blocks on all chromosomes. In addition, *Proceratophrys boiei* females have a metacentric W chromosome showing heterochromatin in all chromosomal extension defined by the C-banding technique, e.g., C-positive blocks, being the W chromosome smaller than Z in females (Ananias et al., 2007; Amaro et al., 2012; Da Silva et al., 2020; Silva et al., 2021). So far, it is the only specie of the genus with these atypical chromosomal characteristics, even for amphibians. However, for different populations of *P. boiei*, significant cytogenetic differences have already been reported, mainly in relation to the number of heterochromatic blocks in the chromosomes and the presence of the differentiated sex chromosome W (Amaro et al., 2012; Da Silva et al., 2020).

Genomic and cytogenetics analyzes were performed for the first time in a population of *P. boiei* from southern Brazil, which does not have large blocks of heterochromatin and does not have a differentiated W chromosome. We uncovered a large number of repetitive sequences on its genome, especially satDNAs, possibly involved in heterochromatin formation and maintenance in the species (Da Silva et al., 2020). Now, intrigued by the high amount of C-positive heterochromatin in *P. boiei* from populations of southeastern Brazil and the highly heterochromatic W chromosome in females, we performed here a high-throughput genomic, bioinformatic and cytogenetic analyses to characterize the entire satellite DNA content and complement the satellitome for *P. boiei*, as well as its possible implications for heterochromatin formation and sex chromosome differentiation, and thus carry out the most complete quantification of the satellitome in a frog genome so far.

## 2 Materials and methods

### 2.1 Species sampling, biological materials, and chromosome preparation

Chromosomal preparations and tissue samples from ten males and five females from *P. boiei* were analyzed. The individuals were collected in the wild under collection licenses issued by the Chico Mendes Institute for Biodiversity Conservation (ICMBio) protocol Nos. 70213–1, 70213–2 and 70213–3, in the Brazilian cities of Mogi das Cruzes, state of São Paulo and Camanducaia, state of Minas Gerais. The use of wild animals, as well as the biological tissues used, were registered in the SisGen–National System for the Management of Genetic Heritage and Associated Traditional Knowledge (registration code: AEF54D5). In addition, we used cytogenetic preparations of *P. boiei* already available at the Animal Cytogenetics Laboratory in UNESP, Rio Claro, São Paulo, Brazil, from previous studies (Da Silva et al., 2020; Silva et al., 2021).

Metaphasic chromosomes were obtained from intestinal epithelial cells according to the protocol proposed by Schmid (1978), and the bone marrow and liver were collected according to Baldissera et al. (1993). All procedures for sampling, material handling, and analysis were authorized and approved by the Animal Ethics Committee (Comitê de Ética no Uso de Animais - CEUA - permission 21/2019), Biosciences Institute, UNESP, Rio Claro, SP, Brazil. Finally, the animals were deposited in the Célio F. B. Haddad (CFBH) amphibian collection, housed in the Department of Biodiversity, Biosciences Institute, UNESP, Rio Claro, SP, Brazil.

### 2.2 Genomic DNA extraction, genome sequencing, and satellitome analysis

Genomic DNA (gDNA) extraction was obtained from liver or muscle samples using the Wizard^®^ Genomic DNA purification kit (Promega, WI, United States), according to the manufacturer’s recommendations. This gDNA was later used for genomic sequencing and polymerase chain reaction (PCR) assays. One individual of each sex was used for genome paired-end sequencing (2 × 101 bp) through Illumina^®^ Hiseq™ 2000 by Macrogen Inc. (Seoul, Republic of Korea).

From sequenced libraries of both female and male individual, satDNAs sequences were recovered using different approaches for a complete search for the satellitome of the species. In addition, a comparative approach was given, focusing on the possible differences between the sexes, in search of satDNAs that could be more representative in the female genome and probably enriched in the heteromorphic sex chromosome W.

To perform a high-throughput analysis, the satMiner bioinformatics protocol for satDNA prospection in both libraries was used (Ruiz-Ruano et al., 2016), available at GitHub (https://github.com/fjruizruano/satminer, accessed on 1 April 2021). The satMiner protocol uses several rounds of clustering in RepeatExplorer (RE) (Novák et al., 2013) and most recently RepeatExplorer2 (Novák et al., 2020) to identify and extract satDNA sequences, and each round includes filtering out reads matching previously assembled contigs with deconseq 0.4.3 (Schmieder and Edwards, 2011), in order to identify and extract as many repetitive sequences as possible, even with low abundance in the genome. It started with a library sampling of 200,000 reads, incrementing this number by two in each consequent round of RE clustering.

RE clusters putatively containing satDNAs were selected for each round by visual graph inspection to identify spherical or ring shapes which are characteristic of this type of tandem DNA sequence. Each cluster was manually analyzed for their internal contigs structure and tandem repetitions were investigated using the dotplot tool implemented in Geneious v4.8 (Drummond et al., 2015) and Tandem Repeats Finder (TRF) (https://tandem.bu.edu/trf/trf.html, accessed on 1 April 2021) (Benson, 1999). In addition, for each run, the output generated by the TAREAN tool (Novák et al., 2017) coupled to the RE for automatic identification of satDNAs was analyzed, with the same parameters and definitions for each run in the RE, and all possible satellite DNAs, with high or low reliability, were considered and analyzed manually, integrating the final satellitome. The clustering and filtering steps were repeated six times for the female and male libraries, adding new filtered reads in each iteration until we could no longer detect new satDNAs in neither.

The satDNAs consensus were compared to search for homology using multiple sequence alignments with Muscle (Edgar, 2004) implemented in Geneious v4.8 software (Drummond et al., 2015) and running a homology test based on RepeatMasker (Smit et al., 2013) with “rm_homology.py” (https://github.com/fjruizruano/ngs-protocols, accessed on 1 April 2021). The results of these analyses were used to classify the satDNA collection into superfamilies, families and/or subfamilies, and all satDNA families were numbered in order of decreasing abundance in the female genome, following the identity criterion proposed by Ruiz-Ruano et al. (2016).

Also, searches were performed for each satellite DNA family using the Censor tool (http://www.girinst.org/, accessed on 1 April 2022) against Repbases. Furthermore, in satMiner analyses, the RepeatMasker tool is already coupled, which automatically searches for similarities with possible transposable elements and other sequences deposited in this database. Then, we searched all databases for any similarities to satDNAs consensus sequences using the BLASTN tool (https://blast.ncbi.nlm.nih.gov/Blast.cgi, accessed on 1 April 2022). We BLAST-searched specially for satDNAs previously detected and deposited for *P. boiei* (Da Silva et al., 2020), to check the presence of conserved satDNAs in different populations for this species. All consensus sequences of the satDNAs characterized in this work are deposited in GenBank (NCBI), under accession numbers OP223503 - OP223728.

### 2.3 Estimation of SatDNAs sequence abundances and divergences in the genome

RepeatMasker (Smit et al., 2013) with rmblast engine was used to determine abundance and average nucleotide divergence (Kimura-2- parameter, K2P) for each satDNA family in both sexes. Genomic abundance for every satDNA in the male and female libraries was estimated as the number of nucleotides aligned to the reference consensus divided by the library size (in bp). With this data repeat landscapes were generated for the relative abundance (*Y*-axis) at 1% intervals of K2P distance from the consensus (*X*-axis), using the script calcDivergencFromAlign.pl (from RepeatMasker utils). A subtractive landscape was subsequently generated to evaluate which satDNA families differ between both libraries to provide the first indications of which satDNA are more prominent in one sex in comparison to the other. All landscapes graphics were built using R programming (R Core Team, 2021 - version 4.1.2).

The different enrichment of all satDNAs across the sexes was determined by generating a female to male ratio as we calculated the quotient between the abundance values of each satDNA family. This data complemented the subtractive landscape by providing more between-sexes differences, as satDNA families with Female/Male (F/M) ratio higher than one was considered more abundant in females (as the threshold to determine it as more prevalent in this sex). The 59 satDNA families that are most enriched in each sex considering the F/M ratio were selected for profiling and we generated individual landscapes for each selected female-biased satDNAs to confirm different amplification and divergence between the sexes.

### 2.4 DNA amplification and chromosomal mapping of repetitive DNAs

Primers were manually designed for the ten most abundant satDNA families in *P. boiei* and manufactured by Exxtend Biotechnology Ltd. (Paulínia, São Paulo, Brazil). The PCR conditions for these sequences followed the same protocol described in Da Silva et al. (2020). All amplified sequences were sequenced by the Sanger method to confirm their actual amplification.

Fluorescence *in situ* hybridization (FISH) was performed on mitotic chromosome spreads from adults using one or two probes simultaneously. With the exception of PboSat03-25, which was labelled with biotin-14-dATP (Invitrogen^®^) at 5′end during its synthesis, the sequences of each satDNA obtained through PCR were labeled by nick-translation with digoxigenin-11-dUTP (Roche^®^) and detected by antidigoxigenin-rhodamine (Roche); PboSat03-25 sequences were detected by Alexa Fluor 488-conjugated (Invitrogen), following the method previously described by Pinkel et al. (1986), with adjustments described by Da Silva et al. (2020) and Cabral-de-Mello and Marec (2021).

The chromosomes were counterstained using 4′,6-diamidine-20-phenylindole dihydrochloride (DAPI) and slides were mounted in VECTASHIELD (Vector, Burlingame, CA, United States). The resulting slides were visualized under an Olympus^®^ BX51 fluorescence microscope, with a digital camera Olympus^®^ DP71 attached, and the images were captured using the DP Controller camera software. For each slide, a minimum of 10 metaphases were analyzed and photographed to confirm the FISH results.

## 3 Results

### 3.1 High-throughput analysis of the satellitome

The female library sequencing provided about 2.0 Gb of sequence data (1,957,610,886 reads), yielded 19,382,286 paired-end trimmed reads and for the male library, about 1.4 Gb of sequence data (1,446,983,166 reads) and 14,326,566 paired-end trimmed reads. The six iterations performed by the satMiner protocol on male and female genome of *P. boiei* uncovered 226 different satDNA families for both sexes. The predominance of repeat unit lengths (RUL) ranging from 20 to 986 bp (average of 69 bp) and the total satDNA abundance comprised 16.87% of *P. boiei* genome, with abundance per family ranging from 0.00002% to 10.49%.

The A+ T content of consensus satDNA sequences varied between 29.3% and 76.9% (55% on average), which indicated a slight bias towards A+ T rich satellites (Supplementary Table S1). Homology tests between all satDNA families revealed the occurrence of 15 superfamilies (SFs), with homologies between 50.2% and 80%, and as expected, the families belonging to each SF showed highly similar sequence properties (RUL and A+ T content), as the superfamilies 03, 08, 09, 10, 11, 12, and 15, in which they have sequences with similar characteristics (Supplementary Figure S1).

Interestingly, PboSat01-176 comprises 93.62% of the total amount of satDNAs families, being the most abundant satDNA in the genome of *P. boiei*, comprising 10.49% of genomic abundance. The second most abundant satDNA corresponds only to 0.97% of total satDNA content and all other satDNAs are in very low abundances. The values determined by RepeatMasker for divergence were relatively variable for the species as a whole, ranging from 1.93% to 24.01% (average divergence for the species was 10.96%).

For PboSat01-176 and PboSat02-192, the genomic abundance in the present work refers to the values determined by the RepeatMasker which applied a substantial number of reads compared to the set of randomly selected reads previously analyzed for another population of *P. boiei* (and only *via* the RepeatExplorer output) by Da Silva et al. (2020). Thus, PboSat01-176 remained the most abundant satDNA family, also, PboSat02-192 (formerly PboSat03-189) came in second in abundance after this thorough analysis, and the previously described satDNA PboSat02-173 was discarded, as it was highly similar to PboSat01-176, redescribed in the present work.

As expected, GenBank searches resulted in similarities with some families of satDNAs previously deposited for *P. boiei* by Da Silva et al. (2020). BLAST results and subsequent alignments for other satDNAs showed high and low similarities to other repetitive sequences, mainly microsatellites, from other related organisms. The search in Censor Repbase showed that some consensus sequences of satDNA families share a certain degree of similarity with transposable elements, among other sequences; however, all with non-significant sequence identity. Also, most satDNA families, 144 in total, does not show any similarity with transposable elements. However, surprisingly, 82 families show some similarity, low or high, with retrotransposons, mainly Ty3/Gypsy and Tc/Mariner (Supplementary Table S1). Similarities with other types of retrotransposons were also found, as well as DNA transposons.

### 3.2 Satellitome differences between the sexes

**FIGURE 1:**
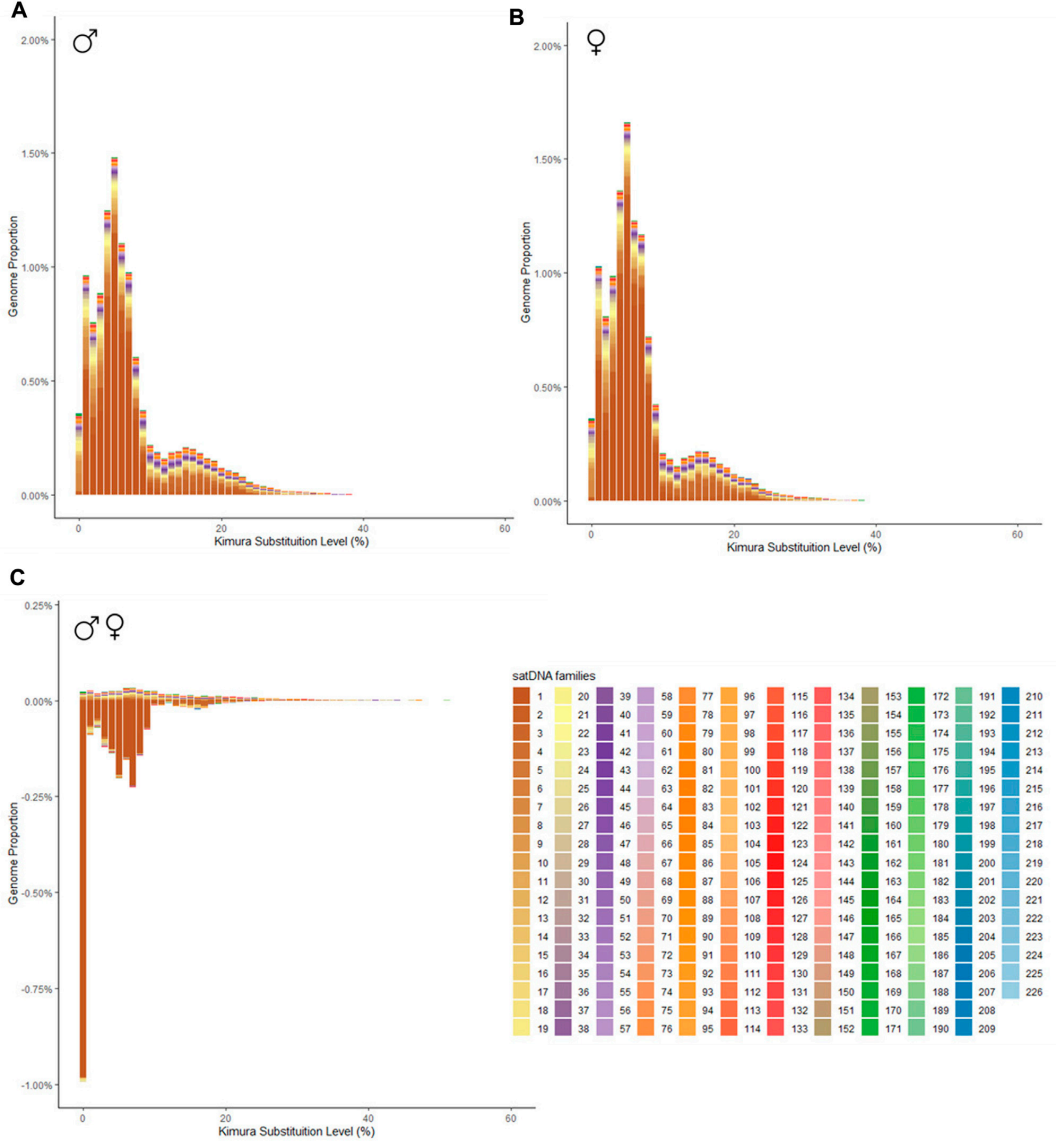
Repeat landscape (abundance vs divergence) for satDNAs identified in *Proceratophrys boiei*. The graphs show, for each color-coded element, the sequence divergence according to Kimura distance (*x*-axis) in relation to their copies in the genome (*y*-axis). **(A)** male genome; **(B)** female genome; **(C)** Subtractive repetitive landscape. The graphs show, for each color-coded element, the sequence divergence according to Kimura distance (*x*-axis) in relation to their copies in the genome (*y*-axis). Copies clustered to the left (lower divergence) potentially correspond to recent copies occurring in the genome. In the subtractive graphic, abundance values show the difference between the male minus the female genomes, thus, positive values indicate overabundance in the male, and negative values indicate overabundance in the female genome.

Bioinformatic analysis revealed that all 226 satDNA families found in the *P. boiei* genome are shared between both sexes. These, however, are differently enriched between them, 59 satDNAs had a F/M ratio greater than 1, suggesting an enrichment in the female library, while 159 were considered male (F/M ratio less than 1), and, eight of them had an F/M ratio equal to 1, having the same abundance on both genomes. To show differences in satellitome between the sexes, individual repeat landscapes for male and female were generated (Figures 1A, B, respectively), in addition to a subtractive landscape (Figure 1C), comparing the two genomes.

**FIGURE 2:**
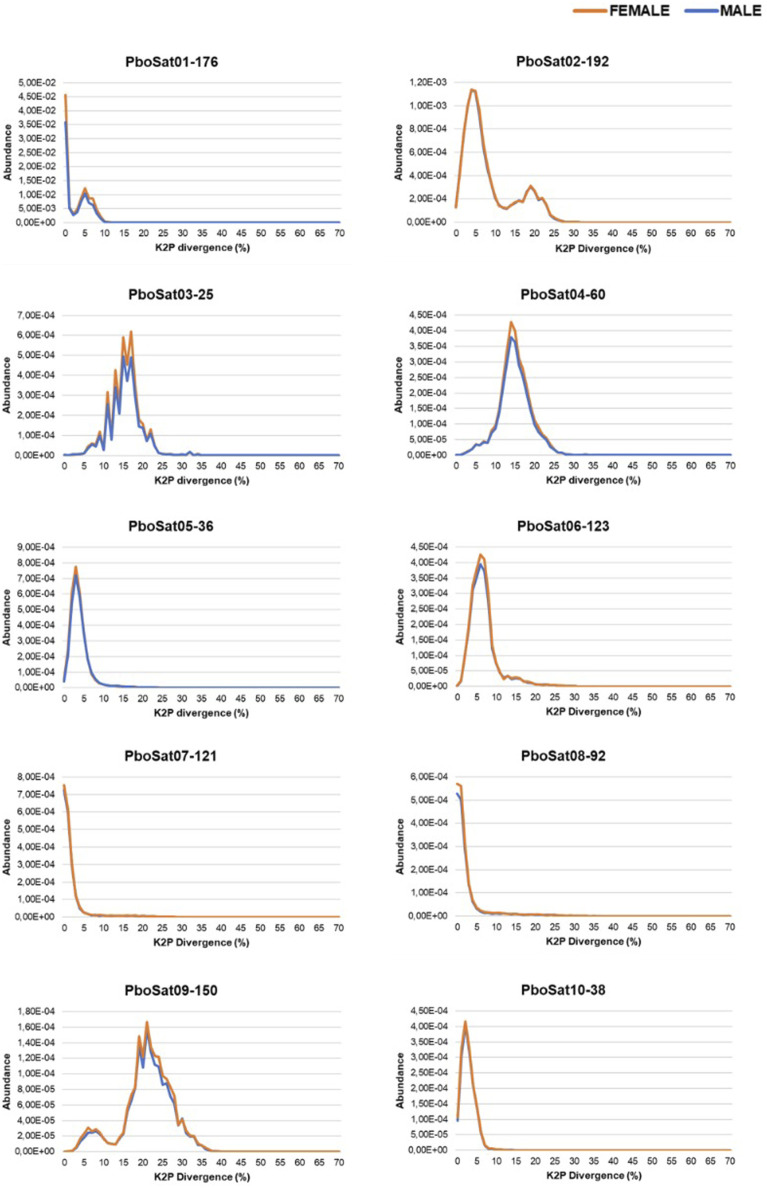
Individual satDNA landscapes of male (blue) and female (orange) repeats for ten more abundant satDNAs families identified in the *Proceratophrys boiei* genome.

In order to verify families of satDNAs that could be highly biased towards females, the F/M ratio revealed 59 satDNAs biased for the female library and, consequently, putatively clustered on the W sex chromosome. To demonstrate the abundance and divergence for the ten most abundant satDNA families identified in the genome of Proceratophrys boiei, individual landscapes were constructed, showing an evolutionary dynamic for each satDNA repeat analyzed (Figure 2). However, the total composition of the satellitome in *P. boiei* corresponded to 16.87% and 14.91% of the female and male genomes, respectively. Almost all satDNAs showed very similar divergence values for both males and females, with exception of PboSat221-28 (23.86% and 17.88%, for female and male respectively), PboSat223-28 (19.54% and 16.16%), PboSat224-38 (30.32% and 21.89%), PboSat225-31 (13.86% and 16.76%), and PboSat226-20 (14.52% and 18.4%), interestingly, the least abundant satDNAs (Supplementary Table S1).

### 3.3 Chromosome mapping of the most abundant SatDNAs families

**FIGURE 3:**
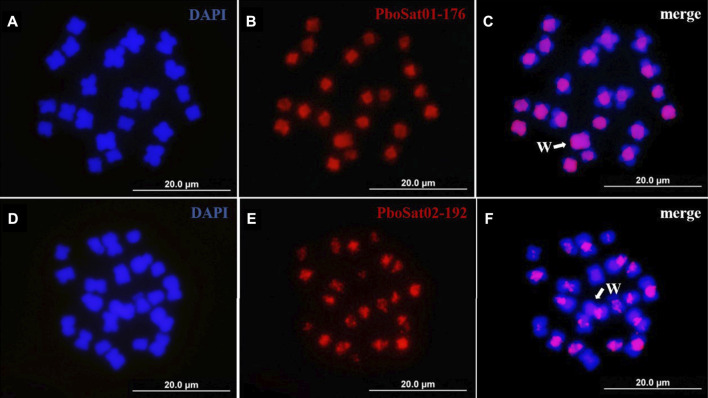
FISH mapping of two more abundant satDNAs (red) and in mitotic metaphase of Proceratophrys boiei female individual. **(A,D)** chromosomes counterstained with DAPI; **(B)** PboSat01-176; **(E)** PboSat02-192; **(C,F)** merge. Note that both satDNAs showed a high clustering in centromeric and pericentromeric region of chromosomes. Sex chromosome W is highlighting in C and F (arrows).

The physical mapping by FISH on female and male chromosomes of *P. boiei* for the ten most abundant satDNAs families was performed to study the organization of those families in the genome and analyze whether the genomic results reflect at the cytogenetic level. FISH was performed for ten most abundant satellites; however, satisfactory hybridization results were only found for the satellites PboSat01-176 and PboSat02-192, with visible signs of clustering on chromosomes (Figure 3).

The chromosomal locations for these satDNAs families indicate that the FISH bands found in the female and male *P. boiei* chromosomes were located in pericentromeric regions surrounding the centromeres. It is evident that the pericentric heterochromatic regions are enriched in satDNA in *P. boiei*, as they contained the most abundant first families, representing 81% of all satDNA content in the species genome (Supplementary Table S1). It was expected that the most abundant satellites in the female genome would be found by FISH on the W sex chromosome; however, none of them was found exclusively on this chromosome.

## 4 Discussion

The complete satellitome of *P. boiei* provided in this work is composed of a high number of satellite families. The in-depth characterization of 226 satDNAs makes *P. boiei* the frog species with the highest number of satellites described so far. Similarity analyzes showed that 67 of the satDNAs found can be grouped into 15 superfamilies. Notably, satDNA sequences are considered crucial factors in genomic and karyotypic evolution, perhaps most importantly, they may constitute rapidly evolving genome sequences (Garrido-Ramos, 2017; Ahmad et al., 2020).

By performing several successive filtering steps and searches with satMiner and RepeatExplorer, in each step subtracting those repetitive elements found in previous steps, the chance of finding other poorly represented satDNAs is substantially increased (Ruiz-Ruano et al., 2016). This genomic mining approach has been used efficiently for the analysis of TEs and satDNA content in distinct species in the last few years, e.g., plants, insects and fish, providing an opportunity to uncover satDNA families whose isolation was elusive by other methods (Ruiz-Ruano et al., 2018; Utsunomia et al., 2019; Crepaldi et al., 2021; Camacho et al., 2022). In this sense, the discovery of a large number of satellite families, with several of them grouped into superfamilies, draws attention to an expansion of satDNAs through duplications of existing repeats, followed by substitutions/deletions/insertion events in the genome of *P. boiei* (Ruiz-Ruano et al., 2016; Utsunomia et al., 2019). In previous analyzes by Da Silva et al. (2020), using the methodology available at the time, the authors found a large amount of satDNAs from another population for *P. boiei*. However, in the more accurate analyzes carried out in this work, the previous data are contrasted and complemented, revealing the need to update the genomic content data of satDNAs for *P. boiei*.

Studies on repetitive DNA, and particularly on satDNA, are of high interest to better understand genome structure and dynamics (Ruiz-Ruano et al., 2018). In this work, valuable information was retrieved regarding the evolutionary dynamics of these sequences and their genomic organization in *P. boiei* frog. The fact that the satellitome presents as mostly small and highly diversified sequences, with low divergence, suggests that recent amplification and diversification events occurred in the *P. boiei* genome, which may have followed the heterochromatinization events as evidenced by C-banding (Ananias et al., 2007; Amaro et al., 2012; Da Silva et al., 2020). In terms of recent diversification of satDNA sequences, this is well shown for fish, such as *Astyanax* (Silva et al., 2017), *Megaleporinus macrocephalus* (Utsunomia et al., 2019), *Characidium gomesi* (Serrano-Freitas et al., 2020) and *Megaleporinus elongatus* (Crepaldi et al., 2021), making it clear that evolutionary mechanisms for these repetitive sequences behave similarly in distinct groups of organisms.

It is well known that novel families of satDNAs can arise from the independent duplication of different genomic sequences such as intergenic spacers, portions of transposable elements, or even those derived from other satellite DNAs (Ruiz-Ruano et al., 2016; Garrido-Ramos, 2017; Ahmad et al., 2020; Thakur et al., 2021). About satDNAs grouped into superfamilies in the *P. boiei* genome, it is remarkable that these satellites actually belong to sequence groups and may be subject to similar genome duplication and amplification, as they have common characteristics, such as monomer size and low genomic abundance. More studies are needed to understand the evolutive dynamics specifically of these repetitions.

Consistent with its richness of centromeric C heterochromatin, it became apparent that the genome of *P. boiei* is enriched in high copy repetitive DNAs. However, in relation to the highly heterochromatic W sex chromosome, unlike expected, specific satDNAs to this chromosome were not evidenced, leading to the belief that there was still no clustering of repeats directly related to the differentiation of sex chromosomes in this species. We suggest that this W chromosome in females of *P. boiei* may be a young element currently in the initial phase of heterochromatinization and differentiation. Our bioinformatics/FISH analyzes showed that the absence of sex-specific signals may be due to negligible absence, loss or gain, or due to non-differentiation of the DNA content of the sex chromosomes in *P. boiei*. Although the FISH technique has limitations for detecting less abundant sequences, this may still suggest an early stage of differentiation of the content of the ZZ/ZW sex chromosome system in *P. boiei*, with no evident molecular differentiation between the heteromorphic sex chromosomes.

As expected, strategic mapping by FISH for certain sequences revealed that most satDNAs were not detectable by the technique in either sex, since in previous analyzes it was also not detectable, especially those satDNAs with very low abundance. In high-throughput analysis, it has been easy to characterize a large amount of satDNAs; however, to show positive signals in FISH, there is some difficulty, mainly due to the low abundance and the difficulty in performing the technique (Ruiz-Ruano et al., 2016; Silva et al., 2017; Cabral-de-Mello and Marec, 2021; Crepaldi et al., 2021). Also, the majority of the satellitome already described is arranged in small arrays below the detection threshold of FISH, and for the *P. boiei* satellitome analyzed here it is no different (Ruiz-Ruano et al., 2016; Bardella et al., 2020; Ferretti et al., 2020; Crepaldi et al., 2021). Thus, in this work we can suggest that the detection of low-abundant satDNAs signals in amphibian genomes should be improved with adaptations of FISH protocols and more specific experiments, which could lead to a more accurate knowledge of these less abundant genomic repeats.

For the highly abundant PboSat01-176 and PboSat02-192 satellites, the successful mapping by FISH highlights the presence of certain satDNAs in strategic regions of the chromosomes of *P. boiei* (e.g., centromere and pericentromeric regions), which leads to their supposedly part in crucial processes for genomic organization and maintenance in this species. It is well highlighted that the abundance of satDNAs is prone to change rapidly due to evolutionary molecular mechanisms such as scattering and amplification, and can result in rapid repatterning as they expand or shrink their arrays (Plohl et al., 2008; Garrido-Ramos, 2017; Palacios-Gimenez et al., 2020; Sproul et al., 2020; Thakur et al., 2021); this rapid evolution is probably also occurring in *P. boiei*. In amphibians, abundant repeats of satDNAs seem not to be dispersed in the karyotype, and even in specific chromosomes, but rather clustered in peri/centromeric regions, and possibly playing a role in the organization, regulation and maintenance of these chromosomal regions. In summary, this feature was detailed for the anuran genera *Physalaemus*, *Proceratophrys*, and *Bufo* (Vittorazzi et al., 2011; Da Silva et al., 2020; Guzmán et al., 2022, respectively), and, for all cases, the chromosomal location of abundant satDNAs indicates a role in centromere function or in the formation and maintenance of heterochromatin in these regions.

Previously, our selection of satDNAs *via* analysis of relative abundance values (female/male) revealed the presence of 58 satDNAs that possible had differentially accumulated within the heteromorphic sex chromosomes of *P. boiei*. We show here a great diversity of satellites in the genome of *P. boiei*; however, this high amplification does not seem to be the main factor that leads to the heterochromatic expansion of the W chromosome, since none of the satDNAs was mapped exclusively on this chromosome, being always shared equally between the both chromosomes pairs. Unlike the fish *M. elongatus*, in which high rates of amplification and homogenization of some satDNAs particularly abundant in the female genome effectively contributed to the heterogametic differentiation of the sex chromosomes of this species (Crepaldi et al., 2021).

Different studies have already shown that TEs are abundant in sex chromosomes. TEs may play a significant role in sex chromosome differentiation by allowing W/Y chromosomes to achieve a state of beneficial non-homology/non-recombination *via* TE insertions in a brief time (McDonald, 1993; Charlesworth and Charlesworth, 2000; Hua-Van et al., 2005; Bachtrog, 2008). Here, surprisingly, it was found that of the 226 satellite families found for *P. boiei*, 82 of them show some similarity, low or high, with retrotransposons, mainly Ty3/Gypsy and Tc/Mariner, being an indication of plasticity and mobility of these repetitive elements for the organization, regulation, and enlargement of *P. boiei* genome. As mobile elements, TEs can replicate and spread within genomes through transposition, leading to an increase in copy number by intrinsic replication, as in the case of retrotransposons, or the repair of double-strand breaks generated during transposition (Eickbush and Malik, 2002).

The insertion of TEs has been postulated to be one of the earliest triggers causing recombination suppression (Kent et al., 2017; Drost and Sanchez, 2019). Although a more in-depth analysis of this relationship in *P. boiei* has not been done, it is notable that TEs are driving genomic functions in this species. Whether plant and animal sex chromosomes preferentially accumulate specific TEs compared to the other chromosomes remains unclear. This suggests that the non-random distribution of Ty3/Gypsy in the genome may drive sex chromosome differentiation. However, investigating the possible existence of Ty3/Gypsy elements in other organisms, using fluorescence *in situ* hybridization mapping, bioinformatics analysis, and whole-genome sequencing may further substantiate this hypothesis, particularly in *P. boiei*.

In summary, our study reveals the complete satellitome of *P. boiei*, showing a great diversity of repeats that are driving genomic organization in this frog species. The characterization and approaches regarding satDNAs in this species of frog allowed the confirmation of some insights from satellite biology and a possible relationship about the evolution of sex chromosomes, especially in anuran amphibians, which did not yet have complete data from this species so far. When combined with future analyses, these data will be useful for the accurate characterization of specific satellites, as well as transposable elements, their relationship with centromeric functions, and their possible influence on sex chromosome differentiation in *Proceratophrys*.

## Data Availability

The datasets presented in this study can be found in online repositories. The names of the repository/repositories and accession number(s) can be found in the article/Supplementary Material.
