# Supplementary material for: Analysis in Proceratophrys boiei genome illuminates the satellite DNA content in a frog from the Brazilian Atlantic forest
Source: Front Genet. 2023 Mar 29;14:1101397. doi: 10.3389/fgene.2023.1101397 (PMC10095563; doi:10.3389/fgene.2023.1101397)
Supplement: Supplementary file 1 [file Table1.DOCX]

Supplementary Material

#
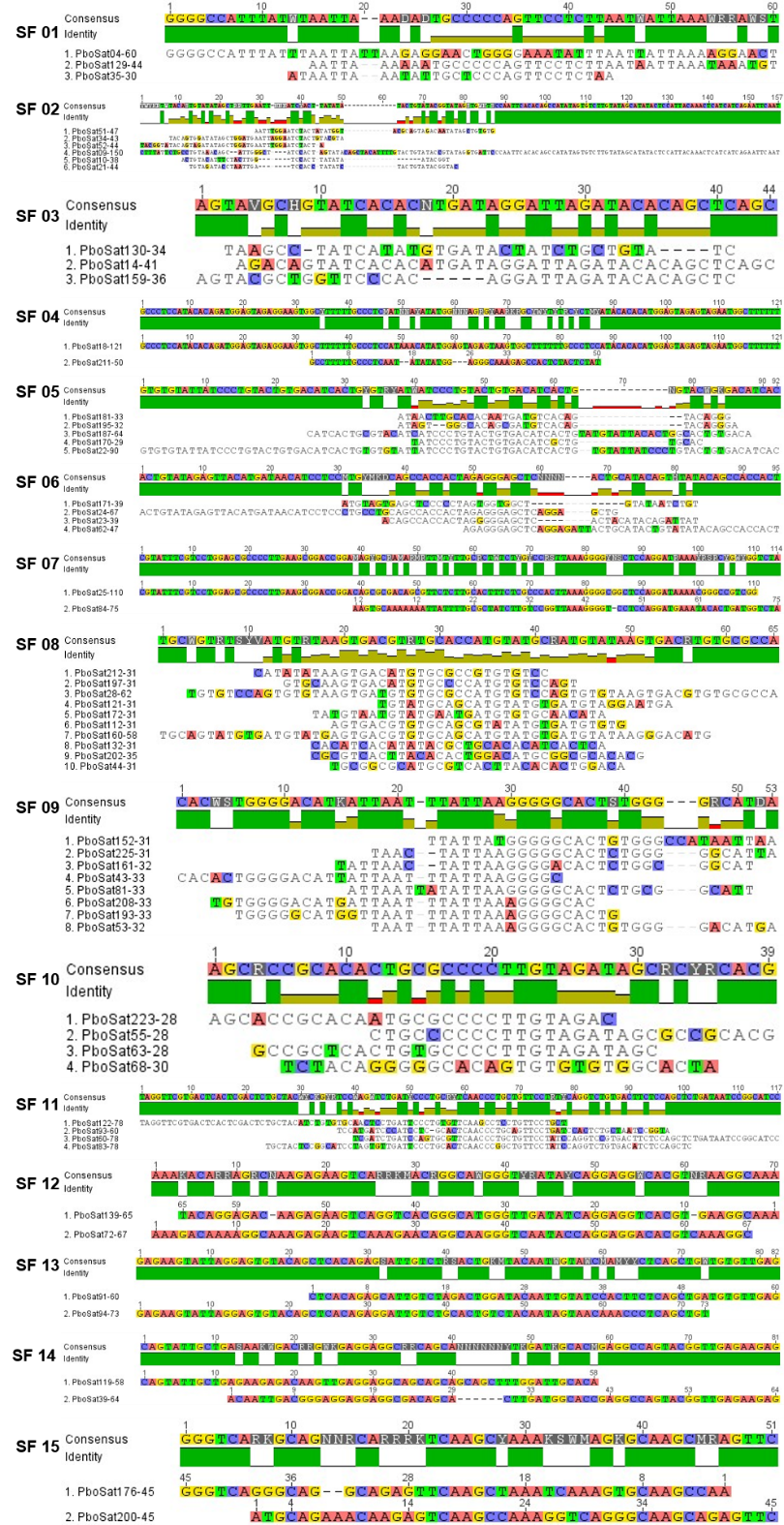
Supplementary Figures and Tables

**Supplementary Figure S1**. Alignments between sequences from the characterized superfamilies in the *Proceratophrys boiei* genome.

| SF | Satellite DNA family | Length | A+T | Abundance | | Divergence | | F/M |
| --- | --- | --- | --- | --- | --- | --- | --- | --- |
|  |  |  |  | **F** | **M** | **F** | **M** |  |
|  | PboSat01-176 | 176 | 54,5 | 10,4973 | 8,4003 | 3,10 | 3,08 | 1,25 |
|  | PboSat02-192 | 192 | 53,6 | 0,9736 | 0,9894 | 8,61 | 8,65 | 0,98 |
|  | PboSat03-25 | 25 | 48,0 | 0,4102 | 0,3421 | 15,98 | 16,01 | 1,20 |
| 01 | PboSat04-60 | 60 | 71,6 | 0,3294 | 0,2975 | 15,42 | 15,40 | 1,11 |
|  | PboSat05-36 | 36 | 67,7 | 0,3123 | 0,2932 | 4,0 | 4,15 | 1,06 |
|  | PboSat06-123* | 123 | 64,3 | 0,2463 | 0,2634 | 7,12 | 7,13 | 0,93 |
|  | PboSat07-121* | 121 | 71,1 | 0,1961 | 0,2040 | 2,05 | 2,10 | 0,96 |
|  | PboSat08-92 | 92 | 57,6 | 0,1743 | 0,1913 | 2,85 | 2,86 | 0,91 |
| 02 | PboSat09-150 | 150 | 63,3 | 0,1635 | 0,1786 | 21,42 | 21,39 | 0,92 |
| 02 | PboSat10-38 | 38 | 65,8 | 0,1557 | 0,1624 | 2,76 | 2,74 | 0,96 |
| 03 | PboSat11-72* | 72 | 66,7 | 0,1427 | 0,1667 | 9,46 | 9,84 | 0,86 |
|  | PboSat12-52 | 52 | 50,9 | 0,1313 | 0,1479 | 7,56 | 7,65 | 0,89 |
|  | PboSat13-181 | 181 | 29,3 | 0,1162 | 0,1459 | 8,95 | 9,39 | 0,80 |
| 04 | PboSat14-41 | 41 | 58,3 | 0,1155 | 0,1254 | 6,03 | 6,42 | 0,92 |
|  | PboSat15-166 | 166 | 38,0 | 0,1118 | 0,0883 | 3,26 | 3,45 | 1,27 |
|  | PboSat16-25* | 25 | 52,0 | 0,1063 | 0,0873 | 15,68 | 15,74 | 1,22 |
|  | PboSat17-90 | 90 | 50,0 | 0,1057 | 0,1084 | 5,71 | 5,75 | 0,97 |
| 05 | PboSat18-121 | 121 | 56,2 | 0,0971 | 0,0884 | 11,98 | 11,98 | 1,10 |
|  | PboSat19-178 | 178 | 52,2 | 0,0931 | 0,0995 | 1,90 | 2,01 | 0,94 |
|  | PboSat20-52 | 52 | 47,1 | 0,0855 | 0,0971 | 7,60 | 7,60 | 0,88 |
| 02 | PboSat21-44* | 44 | 63,6 | 0,0853 | 0,0918 | 6,92 | 6,91 | 0,93 |
| 06 | PboSat22-90 | 90 | 57,8 | 0,0827 | 0,0891 | 9,12 | 9,10 | 0,93 |
| 07 | PboSat23-39 | 39 | 51,2 | 0,0753 | 0,0716 | 4,86 | 5,07 | 1,05 |
| 07 | PboSat24-67 | 67 | 47,8 | 0,0746 | 0,0776 | 6,23 | 6,28 | 0,96 |
| 08 | PboSat25-110 | 110 | 38,2 | 0,0733 | 0,0580 | 2,46 | 3,03 | 1,26 |
|  | PboSat26-115 | 115 | 60,7 | 0,0680 | 0,0706 | 8,28 | 8,29 | 1,04 |
|  | PboSat27-24* | 24 | 62,5 | 0,0507 | 0,0541 | 26,88 | 26,75 | 0,94 |
| 09 | PboSat28-62 | 62 | 46,7 | 0,0499 | 0,0573 | 8,66 | 8,70 | 0,87 |
|  | PboSat29-68 | 68 | 73,5 | 0,0473 | 0,0237 | 2,74 | 2,48 | 2,0 |
|  | PboSat30-91 | 91 | 69,3 | 0,0464 | 0,0486 | 2,25 | 2,25 | 0,96 |
|  | PboSat31-33 | 33 | 57,5 | 0,0419 | 0,0420 | 11,45 | 11,55 | 1,0 |
|  | PboSat32-43* | 43 | 58,2 | 0,0407 | 0,0446 | 4,63 | 4,91 | 0,91 |
|  | PboSat33-986 | 986 | 67,6 | 0,0390 | 0,0422 | 1,93 | 2,03 | 0,92 |
| 02 | PboSat34-43 | 43 | 62,8 | 0,0374 | 0,0379 | 9,13 | 9,14 | 0,99 |
| 01 | PboSat35-30* | 30 | 69,9 | 0,0358 | 0,0324 | 12,05 | 11,73 | 1,10 |
|  | PboSat36-39 | 39 | 59,0 | 0,0330 | 0,0351 | 10,47 | 10,74 | 0,94 |
|  | PboSat37-31 | 31 | 58,1 | 0,0319 | 0,0341 | 13,78 | 14,01 | 0,94 |
|  | PboSat38-96 | 96 | 33,3 | 0,0311 | 0,0356 | 11,99 | 12,27 | 0,87 |
| 17 | PboSat39-64* | 64 | 42,2 | 0,0311 | 0,0352 | 8,34 | 7,87 | 0,88 |
| 03 | PboSat40-40 | 40 | 55,0 | 0,0296 | 0,0361 | 11,90 | 11,71 | 0,82 |
|  | PboSat41-84* | 84 | 64,3 | 0,0292 | 0,0333 | 20,13 | 20,19 | 0,89 |
|  | PboSat42-51* | 51 | 53,9 | 0,0282 | 0,0293 | 11,04 | 11,09 | 0,96 |
| 10 | PboSat43-33* | 33 | 60,4 | 0,0235 | 0,0235 | 15,13 | 15,02 | 1,0 |
| 09 | PboSat44-31* | 31 | 42,0 | 0,0225 | 0,0252 | 10,01 | 9,94 | 0,89 |
|  | PboSat45-110 | 110 | 60,9 | 0,0214 | 0,0219 | 10,91 | 10,90 | 0,98 |
| 11 | PboSat46-106* | 106 | 48,1 | 0,0202 | 0,0211 | 6,96 | 6,95 | 0,96 |
|  | PboSat47-91 | 91 | 69,3 | 0,0199 | 0,0211 | 1,35 | 1,62 | 0,94 |
|  | PboSat48-93 | 93 | 63,4 | 0,0196 | 0,0205 | 5,29 | 5,31 | 0,96 |
|  | PboSat49-37* | 37 | 54,0 | 0,0195 | 0,0205 | 21,20 | 21,34 | 0,95 |
|  | PboSat50-30 | 30 | 50,0 | 0,0194 | 0,0224 | 14,89 | 15,03 | 0,87 |
| 02 | PboSat51-47 | 47 | 63,8 | 0,0193 | 0,0215 | 8,52 | 8,59 | 0,90 |
| 02 | PboSat52-44* | 44 | 65,9 | 0,0184 | 0,0202 | 9,29 | 9,18 | 0,91 |
| 10 | PboSat53-32* | 32 | 59,3 | 0,0184 | 0,0185 | 19,91 | 19,86 | 1,0 |
|  | PboSat54-39 | 39 | 61,6 | 0,0183 | 0,0182 | 5,94 | 6,50 | 1,0 |
| 12 | PboSat55-28* | 28 | 32,2 | 0,0181 | 0,0223 | 11,02 | 11,88 | 0,81 |
|  | PboSat56-54* | 54 | 59,2 | 0,0180 | 0,0161 | 8,55 | 9,86 | 1,11 |
|  | PboSat57-88 | 88 | 54,6 | 0,0171 | 0,0272 | 11,67 | 11,69 | 0,63 |
|  | PboSat58-75 | 75 | 61,3 | 0,0170 | 0,0163 | 16,29 | 16,64 | 1,04 |
|  | PboSat59-50 | 50 | 52,0 | 0,0160 | 0,0139 | 10,70 | 11,20 | 1,15 |
| 13 | PboSat60-78 | 78 | 46,2 | 0,0157 | 0,0182 | 4,97 | 5,18 | 0,86 |
|  | PboSat61-74 | 74 | 59,4 | 0,0154 | 0,0165 | 4,17 | 4,58 | 0,93 |
| 07 | PboSat62-47 | 47 | 53,2 | 0,0154 | 0,0164 | 7,40 | 7,40 | 0,94 |
| 12 | PboSat63-28* | 28 | 39,3 | 0,0152 | 0,0186 | 11,1 | 11,14 | 0,82 |
|  | PboSat64-29 | 29 | 51,7 | 0,0151 | 0,0160 | 9,50 | 9,30 | 0,94 |
|  | PboSat65-45 | 45 | 53,3 | 0,0150 | 0,0148 | 12,71 | 12,79 | 1,01 |
|  | PboSat66-31 | 31 | 58,1 | 0,0145 | 0,0159 | 21,0 | 21,08 | 0,91 |
|  | PboSat67-44 | 44 | 54,5 | 0,0143 | 0,0142 | 3,95 | 3,85 | 1,01 |
| 12 | PboSat68-30 | 30 | 43,3 | 0,0142 | 0,0154 | 18,97 | 18,74 | 0,92 |
|  | PboSat69-62* | 62 | 59,7 | 0,0138 | 0,0142 | 6,51 | 6,58 | 0,97 |
|  | PboSat70-88* | 88 | 59,1 | 0,0138 | 0,0154 | 3,56 | 3,53 | 0,90 |
|  | PboSat71-46 | 46 | 50,0 | 0,0137 | 0,0139 | 3,56 | 3,97 | 0,99 |
| 14 | PboSat72-67* | 67 | 53,8 | 0,0133 | 0,0150 | 24,75 | 25,14 | 0,89 |
|  | PboSat73-34* | 34 | 57,8 | 0,0132 | 0,0152 | 10,65 | 10,65 | 0,87 |
|  | PboSat74-88* | 88 | 62,5 | 0,0131 | 0,0135 | 13,21 | 12,79 | 0,97 |
|  | PboSat75-43 | 43 | 30,3 | 0,0127 | 0,0143 | 17,12 | 16,98 | 0,89 |
|  | PboSat76-61* | 61 | 55,8 | 0,0126 | 0,0121 | 17,28 | 17,42 | 1,04 |
| 15 | PboSat77-33* | 33 | 42,4 | 0,0125 | 0,0136 | 10,82 | 10,89 | 0,93 |
|  | PboSat78-29 | 29 | 48,2 | 0,0124 | 0,0139 | 11,33 | 11,32 | 0,89 |
|  | PboSat79-69 | 69 | 50,7 | 0,0124 | 0,0159 | 13,71 | 12,75 | 0,78 |
|  | PboSat80-77* | 77 | 58,5 | 0,0122 | 0,0134 | 7,52 | 7,80 | 0,91 |
| 10 | PboSat81-33 | 33 | 60,5 | 0,0122 | 0,0105 | 11,5 | 11,88 | 1,16 |
|  | PboSat82-34* | 34 | 55,9 | 0,0116 | 0,0111 | 8,84 | 8,72 | 1,04 |
| 13 | PboSat83-78 | 78 | 44,9 | 0,0115 | 0,0123 | 23,64 | 23,76 | 0,93 |
| 08 | PboSat84-75 | 75 | 60,0 | 0,0113 | 0,0125 | 13,57 | 13,48 | 0,91 |
|  | PboSat85-24 | 24 | 66,7 | 0,0110 | 0,0123 | 28,02 | 28,35 | 0,92 |
|  | PboSat86-25 | 25 | 48,0 | 0,0110 | 0,0094 | 20,35 | 20,16 | 1,18 |
|  | PboSat87-33* | 33 | 57,6 | 0,0110 | 0,0150 | 10,16 | 12,50 | 0,73 |
|  | PboSat88-45* | 45 | 44,4 | 0,0110 | 0,0118 | 14,77 | 14,82 | 0,93 |
|  | PboSat89-38* | 38 | 60,5 | 0,0104 | 0,0097 | 14,67 | 13,73 | 1,06 |
| 18 | PboSat90-31 | 31 | 51,6 | 0,0103 | 0,0110 | 19,88 | 19,80 | 0,94 |
| 16 | PboSat91-60 | 60 | 56,7 | 0,0101 | 0,0104 | 11,0 | 10,82 | 0,97 |
|  | PboSat92-93 | 93 | 32,2 | 0,0098 | 0,0073 | 0,27 | 0,26 | 1,30 |
| 13 | PboSat93-60 | 60 | 46,7 | 0,0094 | 0,0107 | 11,49 | 11,48 | 0,88 |
| 16 | PboSat94-73 | 73 | 56,2 | 0,0094 | 0,0103 | 18,46 | 18,01 | 0,91 |
|  | PboSat95-44 | 44 | 61,3 | 0,0090 | 0,0088 | 6,52 | 6,65 | 1,02 |
|  | PboSat96-30 | 30 | 56,7 | 0,0090 | 0,0093 | 15,52 | 15,12 | 0,97 |
|  | PboSat97-41 | 41 | 21,9 | 0,0088 | 0,0106 | 14,89 | 14,51 | 0,83 |
|  | PboSat98-30* | 30 | 50,0 | 0,0088 | 0,0101 | 12,78 | 13,30 | 0,87 |
|  | PboSat99-131* | 131 | 61,8 | 0,0086 | 0,0082 | 4,46 | 4,48 | 1,06 |
|  | PboSat100-36 | 36 | 50,0 | 0,0086 | 0,0083 | 21,58 | 21,89 | 1,04 |
|  | PboSat101-351 | 351 | 67,2 | 0,0085 | 0,0135 | 13,96 | 11,95 | 0,63 |
|  | PboSat102-37 | 37 | 64,8 | 0,0084 | 0,0230 | 15,66 | 13,69 | 0,37 |
|  | PboSat103-97 | 97 | 50,5 | 0,0084 | 0,0085 | 10,85 | 10,79 | 0,99 |
|  | PboSat104-84* | 84 | 60,6 | 0,0083 | 0,0089 | 16,02 | 15,12 | 0,94 |
|  | PboSat105-141 | 141 | 62,4 | 0,0082 | 0,0098 | 14,66 | 14,34 | 0,84 |
|  | PboSat106-405 | 405 | 53,8 | 0,0081 | 0,0113 | 5,59 | 5,88 | 0,72 |
|  | PboSat107-34* | 34 | 57,7 | 0,0078 | 0,0080 | 6,54 | 6,56 | 0,98 |
|  | PboSat108-435 | 435 | 56,3 | 0,0078 | 0,0077 | 3,76 | 3,87 | 1,02 |
|  | PboSat109-88 | 88 | 60,3 | 0,0078 | 0,0081 | 15,11 | 14,79 | 0,96 |
|  | PboSat110-742 | 742 | 60,6 | 0,0076 | 0,0090 | 1,34 | 1,12 | 0,84 |
|  | PboSat111-25* | 25 | 48,0 | 0,0076 | 0,0082 | 14,07 | 13,90 | 0,92 |
| 09 | PboSat112-31 | 31 | 51,6 | 0,0075 | 0,0079 | 10,62 | 11,05 | 0,95 |
|  | PboSat113-27* | 27 | 70,3 | 0,0074 | 0,0082 | 4,42 | 4,20 | 0,91 |
|  | PboSat114-27 | 27 | 55,5 | 0,0073 | 0,0077 | 14,26 | 14,69 | 0,94 |
|  | PboSat115-20 | 20 | 70,0 | 0,0073 | 0,0077 | 8,96 | 8,02 | 0,94 |
|  | PboSat116-44 | 44 | 36,3 | 0,0073 | 0,0076 | 4,44 | 4,46 | 0,96 |
|  | PboSat117-543 | 543 | 53,6 | 0,0072 | 0,0063 | 6,13 | 6,82 | 1,13 |
|  | PboSat118-73 | 73 | 57,5 | 0,0071 | 0,0082 | 4,08 | 4,33 | 0,87 |
| 17 | PboSat119-58* | 58 | 50,0 | 0,0067 | 0,0074 | 9,26 | 8,74 | 0,90 |
|  | PboSat120-21* | 21 | 47,7 | 0,0066 | 0,0076 | 7,28 | 6,90 | 0,88 |
| 09 | PboSat121-31* | 31 | 61,3 | 0,0066 | 0,0060 | 6,30 | 6,49 | 1,09 |
| 13 | PboSat122-78 | 78 | 48,7 | 0,0064 | 0,0073 | 21,73 | 22,56 | 0,89 |
|  | PboSat123-45 | 45 | 46,6 | 0,0063 | 0,0067 | 8,01 | 8,34 | 0,94 |
|  | PboSat124-62 | 62 | 74,2 | 0,0063 | 0,0068 | 16,39 | 16,68 | 0,92 |
|  | PboSat125-47 | 47 | 63,8 | 0,0062 | 0,0043 | 7,20 | 7,20 | 1,43 |
|  | PboSat126-52* | 52 | 50,0 | 0,0062 | 0,0068 | 17,88 | 19,27 | 0,91 |
|  | PboSat127-44* | 44 | 45,5 | 0,0061 | 0,0062 | 12,24 | 12,04 | 0,98 |
|  | PboSat128-68* | 68 | 50,4 | 0,0060 | 0,0062 | 8,61 | 9,20 | 0,97 |
| 01 | PboSat129-44 | 44 | 75,0 | 0,0059 | 0,0054 | 8,66 | 8,89 | 1,09 |
| 04 | PboSat130-34* | 34 | 64,7 | 0,0059 | 0,0064 | 21,52 | 22,42 | 0,93 |
|  | PboSat131-41 | 41 | 48,8 | 0,0059 | 0,0073 | 9,28 | 9,68 | 0,81 |
| 09 | PboSat132-31* | 31 | 54,9 | 0,0056 | 0,0066 | 14,35 | 14,89 | 0,84 |
|  | PboSat133-78 | 78 | 43,6 | 0,0054 | 0,0061 | 6,43 | 6,43 | 0,89 |
|  | PboSat134-39 | 39 | 61,5 | 0,0053 | 0,0061 | 15,43 | 15,35 | 0,88 |
|  | PboSat135-68 | 68 | 44,4 | 0,0053 | 0,0060 | 17,84 | 17,93 | 0,88 |
|  | PboSat136-59* | 59 | 40,7 | 0,0053 | 0,0056 | 4,03 | 4,26 | 0,93 |
|  | PboSat137-34 | 34 | 55,9 | 0,0051 | 0,0050 | 11,66 | 11,47 | 1,03 |
|  | PboSat138-29* | 29 | 48,3 | 0,0047 | 0,0049 | 11,69 | 11,72 | 0,95 |
| 14 | PboSat139-65* | 65 | 49,2 | 0,0047 | 0,0047 | 21,78 | 21,97 | 0,99 |
|  | PboSat140-36* | 36 | 50,0 | 0,0044 | 0,0042 | 7,44 | 8,06 | 1,05 |
|  | PboSat141-39* | 39 | 64,1 | 0,0043 | 0,0051 | 11,02 | 11,80 | 0,85 |
|  | PboSat142-60 | 60 | 66,7 | 0,0043 | 0,0040 | 10,80 | 10,95 | 1,07 |
|  | PboSat143-38* | 38 | 43,8 | 0,0041 | 0,0049 | 4,96 | 4,86 | 0,84 |
|  | PboSat144-98 | 98 | 55,1 | 0,0041 | 0,0045 | 8,59 | 8,39 | 0,90 |
|  | PboSat145-33* | 33 | 47,6 | 0,0040 | 0,0035 | 10,51 | 12,12 | 1,12 |
|  | PboSat146-27* | 27 | 51,7 | 0,0039 | 0,0035 | 5,58 | 6,02 | 1,09 |
|  | PboSat147-21 | 21 | 76,2 | 0,0038 | 0,0024 | 7,88 | 10,13 | 1,60 |
|  | PboSat148-22 | 22 | 45,5 | 0,0038 | 0,0048 | 3,85 | 4,04 | 0,81 |
|  | PboSat149-25* | 25 | 48,0 | 0,0038 | 0,0038 | 3,64 | 3,91 | 1,0 |
|  | PboSat150-51* | 51 | 54,9 | 0,0038 | 0,0040 | 12,98 | 12,86 | 0,95 |
|  | PboSat151-52 | 52 | 63,4 | 0,0037 | 0,0028 | 5,78 | 5,81 | 1,30 |
| 10 | PboSat152-31 | 31 | 58,1 | 0,0036 | 0,0039 | 17,68 | 17,16 | 0,92 |
|  | PboSat153-75 | 75 | 80,0 | 0,0035 | 0,0036 | 4,01 | 4,31 | 0,97 |
|  | PboSat154-25* | 25 | 80,0 | 0,0035 | 0,0043 | 8,31 | 7,62 | 0,82 |
|  | PboSat155-20* | 20 | 75,0 | 0,0035 | 0,0050 | 11,04 | 8,86 | 0,71 |
|  | PboSat156-40* | 40 | 67,5 | 0,0035 | 0,0039 | 14,43 | 14,49 | 0,86 |
| 18 | PboSat157-30* | 30 | 50,0 | 0,0035 | 0,0039 | 16,48 | 16,65 | 0,88 |
|  | PboSat158-41 | 41 | 41,5 | 0,0034 | 0,0037 | 13,22 | 12,90 | 0,91 |
| 04 | PboSat159-36* | 36 | 50,0 | 0,0033 | 0,0035 | 19,21 | 19,17 | 0,94 |
| 09 | PboSat160-58 | 58 | 56,9 | 0,0032 | 0,0039 | 13,74 | 13,77 | 0,83 |
| 10 | PboSat161-32 | 32 | 56,2 | 0,0032 | 0,0031 | 11,82 | 12,68 | 1,03 |
|  | PboSat162-87 | 87 | 80,4 | 0,0032 | 0,0032 | 3,34 | 3,32 | 1,0 |
|  | PboSat163-39 | 39 | 51,3 | 0,0031 | 0,0032 | 8,76 | 8,65 | 0,96 |
|  | PboSat164-30 | 30 | 59,0 | 0,0030 | 0,0034 | 14,15 | 13,71 | 0,89 |
|  | PboSat165-452 | 452 | 48,2 | 0,0029 | 0,0056 | 0,35 | 0,27 | 0,53 |
|  | PboSat166-25 | 25 | 80,0 | 0,0028 | 0,0029 | 8,71 | 9,69 | 0,97 |
|  | PboSat167-25 | 25 | 60,0 | 0,0028 | 0,0035 | 10,42 | 10,66 | 0,80 |
|  | PboSat168-77 | 77 | 54,4 | 0,0028 | 0,0047 | 3,17 | 3,27 | 0,59 |
|  | PboSat169-30 | 30 | 70,0 | 0,0027 | 0,0026 | 11,55 | 10,42 | 1,07 |
| 06 | PboSat170-29 | 29 | 48,2 | 0,0026 | 0,0039 | 8,18 | 6,70 | 0,68 |
| 07 | PboSat171-39 | 39 | 51,2 | 0,0026 | 0,0020 | 4,09 | 4,37 | 1,28 |
| 09 | PboSat172-31 | 31 | 71,0 | 0,0026 | 0,0026 | 17,81 | 18,58 | 0,98 |
|  | PboSat173-29 | 29 | 55,2 | 0,0024 | 0,0024 | 15,59 | 17,20 | 0,99 |
|  | PboSat174-82 | 82 | 59,7 | 0,0024 | 0,0022 | 8,33 | 8,31 | 1,09 |
|  | PboSat175-49 | 49 | 44,9 | 0,0024 | 0,0024 | 19,96 | 19,72 | 1,0 |
| 19 | PboSat176-45* | 45 | 48,9 | 0,0021 | 0,0025 | 21,03 | 21,27 | 0,87 |
|  | PboSat177-75* | 75 | 56,0 | 0,0020 | 0,0016 | 4,78 | 5,21 | 1,23 |
|  | PboSat178-69* | 69 | 58,0 | 0,0019 | 0,0020 | 3,18 | 2,82 | 0,96 |
|  | PboSat179-23 | 23 | 69,5 | 0,0019 | 0,0031 | 11,26 | 11,25 | 0,62 |
|  | PboSat180-42 | 42 | 54,8 | 0,0019 | 0,0023 | 3,14 | 3,27 | 0,84 |
| 06 | PboSat181-33 | 33 | 57,6 | 0,0017 | 0,0020 | 19,85 | 19,12 | 0,84 |
|  | PboSat182-32 | 32 | 43,8 | 0,0016 | 0,0016 | 15,92 | 16,52 | 0,97 |
|  | PboSat183-97 | 97 | 49,5 | 0,0016 | 0,0017 | 9,91 | 10,38 | 0,95 |
|  | PboSat184-39 | 39 | 43,6 | 0,0016 | 0,0017 | 5,58 | 6,05 | 0,94 |
|  | PboSat185-31 | 31 | 58,1 | 0,0015 | 0,0016 | 14,08 | 12,45 | 0,90 |
|  | PboSat186-30 | 30 | 53,4 | 0,0015 | 0,0015 | 5,63 | 6,49 | 0,95 |
| 06 | PboSat187-64 | 64 | 54,7 | 0,0014 | 0,0013 | 18,90 | 20,80 | 1,06 |
| 15 | PboSat188-26 | 26 | 46,1 | 0,0014 | 0,0016 | 12,82 | 10,41 | 0,86 |
|  | PboSat189-20 | 20 | 70,0 | 0,0013 | 0,0014 | 5,42 | 6,39 | 0,98 |
|  | PboSat190-80 | 80 | 60,0 | 0,0013 | 0,0010 | 2,91 | 3,01 | 1,29 |
|  | PboSat191-51 | 51 | 58,8 | 0,0013 | 0,0015 | 11,91 | 12,32 | 0,87 |
|  | PboSat192-30 | 30 | 56,7 | 0,0012 | 0,0014 | 14,46 | 14,11 | 0,87 |
| 10 | PboSat193-33 | 33 | 54,5 | 0,0011 | 0,0012 | 8,05 | 7,99 | 0,92 |
|  | PboSat194-59 | 59 | 40,7 | 0,0011 | 0,0020 | 5,92 | 5,49 | 0,56 |
| 06 | PboSat195-32 | 32 | 46,9 | 0,0011 | 0,0011 | 14,58 | 14,87 | 0,98 |
|  | PboSat196-77* | 77 | 61,1 | 0,0010 | 0,0011 | 11,67 | 12,10 | 0,90 |
| 09 | PboSat197-31 | 31 | 45,2 | 0,0009 | 0,0010 | 17,66 | 17,76 | 0,90 |
|  | PboSat198-44* | 44 | 62,2 | 0,0009 | 0,0008 | 3,16 | 3,02 | 1,08 |
|  | PboSat199-80* | 80 | 55,0 | 0,0009 | 0,0013 | 5,22 | 5,54 | 0,67 |
| 19 | PboSat200-45 | 45 | 51,1 | 0,0009 | 0,0008 | 24,01 | 24,26 | 1,07 |
|  | PboSat201-75 | 75 | 70,6 | 0,0008 | 0,0007 | 0,44 | 0,38 | 1,19 |
| 02 | PboSat202-35 | 35 | 37,1 | 0,0008 | 0,0007 | 10,92 | 11,82 | 1,01 |
|  | PboSat203-52 | 52 | 42,3 | 0,0007 | 0,0010 | 4,98 | 4,45 | 0,78 |
|  | PboSat204-36 | 36 | 72,2 | 0,0007 | 0,0008 | 18,91 | 18,99 | 0,91 |
|  | PboSat205-27 | 27 | 62,9 | 0,0007 | 0,0006 | 6,57 | 7,89 | 1,16 |
|  | PboSat206-69 | 69 | 66,6 | 0,0007 | 0,0006 | 19,67 | 20,13 | 1,10 |
|  | PboSat207-23* | 23 | 64,2 | 0,0007 | 0,0009 | 9,92 | 10,63 | 0,76 |
| 10 | PboSat208-33* | 33 | 60,6 | 0,0006 | 0,0006 | 7,23 | 6,10 | 1,06 |
|  | PboSat209-51 | 51 | 53,0 | 0,0006 | 0,0008 | 19,16 | 22,07 | 0,79 |
|  | PboSat210-30* | 30 | 43,4 | 0,0006 | 0,0005 | 10,66 | 11,24 | 1,10 |
| 05 | PboSat211-50* | 50 | 56,0 | 0,0006 | 0,0005 | 20,68 | 20,57 | 1,17 |
| 09 | PboSat212-31 | 31 | 51,6 | 0,0005 | 0,0006 | 16,74 | 18,61 | 0,80 |
|  | PboSat213-80* | 80 | 58,8 | 0,0004 | 0,0005 | 5,08 | 4,22 | 0,91 |
|  | PboSat214-54* | 54 | 38,9 | 0,0004 | 0,0004 | 7,98 | 8,93 | 1,03 |
|  | PboSat215-25* | 25 | 44,0 | 0,0003 | 0,0003 | 7,76 | 7,87 | 0,99 |
|  | PboSat216-43* | 43 | 34,9 | 0,0003 | 0,0004 | 20,11 | 21,41 | 0,80 |
|  | PboSat217-26* | 26 | 65,4 | 0,0003 | 0,0005 | 16,36 | 16,34 | 0,64 |
|  | PboSat218-25* | 25 | 68,0 | 0,0003 | 0,0003 | 13,82 | 13,39 | 1,0 |
|  | PboSat219-26* | 26 | 76,9 | 0,0002 | 0,0002 | 10,74 | 10,67 | 1,32 |
|  | PboSat220-31* | 31 | 74,2 | 0,0002 | 0,0002 | 19,60 | 17,16 | 1,07 |
| 11 | PboSat221-28* | 28 | 42,8 | 0,0001 | 0,0001 | 17,88 | 23,86 | 0,87 |
|  | PboSat222-29 | 29 | 41,4 | 0,0001 | 0,0002 | 12,51 | 12,43 | 0,60 |
| 12 | PboSat223-28* | 28 | 39,3 | 0,00009 | 0,0001 | 16,16 | 19,54 | 0,89 |
|  | PboSat224-38 | 38 | 52,6 | 0,00009 | 0,00007 | 21,89 | 30,32 | 1,27 |
| 10 | PboSat225-31* | 31 | 51,6 | 0,00008 | 0,0001 | 16,76 | 13,86 | 0,71 |
|  | PboSat226-20* | 20 | 75,0 | 0,00002 | 0,000007 | 18,40 | 14,52 | 2,80 |
|  | **Average** | **98** | **64,75** | **5,24866** | **4,200154** | **10,75** | **8,8** | **2,025** |

**Supplementary Table S1**. General characteristics of satDNA families recovered from male and female genomes of *Proceratophrys boiei*, such as satellite DNA families, length (bp), A+T (%), female (F) and male (M) abundance (% of the genome) and female (F) and male (M) divergence (%). Each satDNA has their own quotient for female and male abundance (F/M ratio). Superfamily (SF) is also evidenced. Averages are at the bottom of the table. *Families of satDNAs with some similarity to a transposable element.

| Primer | Sequence (5’ ^͢^ 3’) |
| --- | --- |
| PboSat01-176F | GTCAGGCGAATGAAAACTC |
| PboSat01-176R | ATTCCTGCACAGAGAAATTG |
| PboSat02-192F | GATTGCGACATTCTACATTGG |
| PboSat02-192R | TACTGCGCCTGTGTATTTGG |
| *PboSat03-25 | ATGGGGGCACAGTGTATATGGCACT |
| PboSat04-60F | CCATTTATTTAATTATTAAGAG |
| PboSat04-60R | CCCCAGTTCCTTTTAATAA |
| PboSat05-36F | TACTATAATACTGCCCCCTA |
| PboSat05-36R | GTTATATTGTTGTAGATAGGG |
| PboSat06-123F | GATTGCGACATTCTACATTGG |
| PboSat06-123R | TACTGCGCCTGTGTATTTGG |
| PboSat07-121F | TAATATATAATGTAATATATAT |
| PboSat07-121R | CATATATACGTTATATATAT |
| PboSat08-92F | CTTATCCTGTACTGATCCT |
| PboSat08-92R | TAATGTATGTACACAGTGAC |
| PboSat09-150F | TGATTCCCAATTCACACAGC |
| PboSat09-150R | CCTATACGGTATACAGTACAA |
| PboSat10-38F | CTTATATAATACGGTACTGT |
| PboSat10-38R | TGGACCAAGTAGAAATGTAC |

*Biotin-labeled primer, no amplification required.

**Supplementary Table S2.** List of primers assigned to the 10 most abundant satellite DNAs for *Proceratophrys boiei*.
